# A multicenter retrospective observational NAPOLEON2 study of nanoliposomal irinotecan with fluorouracil and folinic acid in patients with unresectable pancreatic cancer

**DOI:** 10.1038/s41598-024-63172-y

**Published:** 2024-05-30

**Authors:** Tomoko Kodama, Takashi Imajima, Mototsugu Shimokawa, Taiga Otsuka, Masahiro Kawahira, Junichi Nakazawa, Takeshi Hori, Taro Shibuki, Shiho Arima, Akio Ido, Keisuke Miwa, Yoshinobu Okabe, Futa Koga, Yujiro Ueda, Yoshihito Kubotsu, Hozumi Shimokawa, Shigeyuki Takeshita, Kazuo Nishikawa, Azusa Komori, Satoshi Otsu, Ayumu Hosokawa, Tatsunori Sakai, Kenji Sakai, Hisanobu Oda, Machiko Kawahira, Shuji Arita, Takuya Honda, Hiroki Taguchi, Kengo Tsuneyoshi, Yasunori Kawaguchi, Toshihiro Fujita, Takahiro Sakae, Tsuyoshi Shirakawa, Toshihiko Mizuta, Kenji Mitsugi

**Affiliations:** 1 Department of Medical Oncology, Kagoshima City Hospital, 37-1 Uearata-Cho, Kagoshima-Shi, Kagoshima, 890-8760 Japan; 2 Department of Medical Oncology, Sasebo Kyosai Hospital, 10-17 Shimanji-Cho, Sasebo-Shi, Nagasaki, 857-8575 Japan; 3 Department of Medicine and Biosystemic Science, Kyushu University Graduate School of Medical Sciences, 3-1-1 Maidashi, Higashi‑ku, Fukuoka-Shi, Fukuoka, 812-8582 Japan; 4 Clinical Research Institute, National Kyushu Cancer Center, 3-1-1 Notame, Minami-Ku, Fukuoka-Shi, Fukuoka, 811-1395 Japan; 5 Department of Biostatistics, Yamaguchi University Graduate School of Medicine, 1-1-1 Minamikogushi, Ube-Shi, Yamaguchi, 755-8505 Japan; 6 Department of Internal Medicine, Minato Medical Clinic, 3-11-3 Nagahama, Chuo-Ku, Fukuoka-Shi, Fukuoka, 810-0072 Japan; 7 Department for the Promotion of Drug and Diagnostic Development, Division of Drug and Diagnostic Development Promotion, Translational Research Support Office, National Cancer Center Hospital East, 6-5-1 Kashiwanoha, Kashiwa-Shi, Chiba, 277-8577 Japan; 8 Department of Hepatobiliary and Pancreatic Oncology, National Cancer Center Hospital East, 6-5-1 Kashiwanoha, Kashiwa-Shi, Chiba, 277-8577 Japan; 9 Digestive and Lifestyle Diseases, Kagoshima University Graduate School of Medical and Dental Sciences, 8-35-1 Sakuragaoka, Kagoshima-Shi, Kagoshima, 890-8520 Japan; 10 Multidisciplinary Treatment Cancer Center, Kurume University Hospital, 67 Asahi-Machi, Kurume-Shi, Fukuoka, 830-0011 Japan; 11 Division of Gastroenterology, Department of Medicine, Kurume University School of Medicine, 67 Asahi-Machi, Kurume-Shi, Fukuoka, 830-0011 Japan; 12 Department of Hepatobiliary and Pancreatology, Saga Medical Center Koseikan, 400 Kase-Machi, Saga-Shi, Saga, 840-8571 Japan; 13 Department of Hematology and Oncology, Japanese Red Cross Kumamoto Hospital, 2-1-1 Nagamine-Minami, Higashi-Ku, Kumamoto-Shi, Kumamoto, 861-8520 Japan; 14 Department of Internal Medicine, Karatsu Red Cross Hospital, 2430 Watada, Karatsu-Shi, Saga, 847-8588 Japan; 15 Department of Hematology and Oncology, Japan Community Healthcare Organization Kyushu Hospital, 1-8-1 Kishinoura, Yahatanishi-Ku, Kitakyushu-Shi, Fukuoka, 806-8501 Japan; 16 Department of Gastroenterology, Japanese Red Cross Nagasaki Genbaku Hospital, 3-15 Morimachi, Nagasaki-Shi, Nagasaki, 852-8511 Japan; 17 Department of Medical Oncology and Hematology, Oita University Faculty of Medicine, 1-1 Idaigaoka, Hasama-Machi, Yufu-Shi, Oita, 879-5593 Japan; 18 Department of Gastrointestinal Medical Oncology, National Hospital Organization Shikoku Cancer Center, 160 Kou, Minamiumemoto-Machi, Matsuyama-Shi, Ehime, 791-0280 Japan; 19 Department of Clinical Oncology, University of Miyazaki Hospital, 5200 Kiyotakechoukihara, Miyazaki-Shi, Miyazaki, 889-1692 Japan; 20 Department of Medical Oncology, National Hospital Organization Kumamoto Medical Center, 1-5 Ninomaru, Chuo-Ku, Kumamoto-Shi, Kumamoto, 860-0008 Japan; 21 Department of Clinical Oncology, Japan Community Health Care Organization Hitoyoshi Medical Center, 35 Oikamimachi, Hitoyoshi-Shi, Kumamoto, 868-8555 Japan; 22 Division of Integrative Medical Oncology, Saiseikai Kumamoto Hospital, 5-3-1 Chikami, Minami-Ku, Kumamoto-Shi, Kumamoto, 861-4193 Japan; 23 Department of Gastroenterology, Kagoshima Kouseiren Hospital, 1-13-1 Yojirou, Kagoshima-Shi, Kagoshima, 890-0062 Japan; 24 Department of Chemotherapy, Miyazaki Prefectural Miyazaki Hospital, 5-30 Kita-Takamatsucho, Miyazaki, 880-8510 Japan; 25 Department of Gastroenterology and Hepatology, Nagasaki University Graduate School of Biomedical Sciences, 1-7-1 Sakamoto, Nagasaki-Shi, Nagasaki, 852-8501 Japan; 26 Department of Gastroenterology, Izumi General Medical Center, 520 Myoujin-Cho, Izumi-Shi, Kagoshima, 899-0131 Japan; 27 Department of Gastroenterology, Kagoshima City Hospital, 37-1 Uearata-Cho, Kagoshima-Shi, Kagoshima, 890-8760 Japan; 28 Department of Gastroenterology, Asakura Medical Association Hospital, 422-1 Raiha, Asakura-Shi, Fukuoka, 838-0069 Japan; 29 Department of Gastroenterology, Saiseikai Sendai Hospital, 2-46 Harada-Cho, Satsumasendai-Shi, Kagoshima, 895-0074 Japan; 30 Department of Medical Checkup Center, Eikoh Hospital, 3-8-15 Befu-Nishi, Shime-Machi, Kasuya-Gun, Fukuoka, 811-2232 Japan; 31 Clinical Hematology Oncology Treatment Study Group, 1-14-6 Muromi-Gaoka, Nishi-Ku, Fukuoka-Shi, Fukuoka, 819-0030 Japan; 32 Department of Internal Medicine, Fujikawa Hospital, 1-2-6 Matsubara, Saga-Shi, Saga, 840-0831 Japan

**Keywords:** Nanoliposomal irinotecan, Pancreatic cancer, Second- or later-line, Chemotherapy, Retrospective, Cancer therapy, Pancreatic cancer, Pancreatic cancer, Clinical trial design

## Abstract

Nanoliposomal irinotecan with fluorouracil and folinic acid (NFF) is a standard regimen after gemcitabine-based therapy for patients with unresectable or recurrent pancreatic cancer. However, there are limited clinical data on its efficacy and safety in the real-world. We therefore initiated a retrospective and prospective observational study (NAPOLEON-2). The results of the retrospective part were reported herein. In this retrospective study, we evaluated 161 consecutive patients who received NFF as second-or-later-line regimen. The main endpoint was overall survival (OS), and the other endpoints were response rate, disease control rate, progression-free survival (PFS), dose intensity, and adverse events (AEs). The median age was 67 years (range, 38–85 years). The median OS and PFS were 8.1 and 3.4 months, respectively. The objective response and disease control rates were 5% and 52%, respectively. The median relative dose intensity was 81.6% for nanoliposomal irinotecan and 82.9% for fluorouracil. Grade 3 or 4 hematological and nonhematological AEs occurred in 47 and 42 patients, respectively. Common grade 3 or 4 AEs included neutropenia (24%), anorexia (12%), and leukocytopenia (12%). Subanalysis of patients treated with second-line and third-or-later-line demonstrated no statistical significant difference in OS (7.6 months vs. 9.1 months, respectively; hazard ratio, 0.92; 95% confidence interval, 0.64–1.35; *p* = 0.68). In conclusion, NFF has acceptable efficacy and safety profile even in real-world clinical settings. The prospective study is in progress to validate these findings.

## Introduction

Pancreatic cancer (PC) is one of the deadliest solid cancers, with a 5-year survival rate of < 10%^1–3^, although overall survival (OS) has markedly improved over the past two decades. In Japan, approximately 44,000 new cases of PC were reported in 2019, and 37,677 (18,880 men and 18,797 women) deaths were reported in 2020. Both age-specific morbidity and mortality rates of PC have increased, and the overall 5-year (2014–2015) survival rate is approximately 11.8%^4^, an extremely poor prognosis among malignant tumors.

Most patients with PC have unresectable disease at the time of diagnosis^1^. These patients are not eligible for surgery and are instead recommended for systemic chemotherapy. Gemcitabine (GEM) was initially the standard first-line chemotherapy regimen for patients with advanced PC^5^. However, FOLFIRINOX (FFX; a combination of oxaliplatin, leucovorin, irinotecan, and fluorouracil)^6^ and GEM with nab-paclitaxel (GnP)^7^ have also shown significant survival benefits in patients with metastases and thus become the most common first-line therapy. Despite these benefits, almost all patients develop disease progression, and the 5-year survival rate remains to be poor^1–4^. Hence, the demand for second-line chemotherapy has been increasing. However, although there have been several studies on second-line treatment for advanced PC, the benefits of second-line treatment remain limited^8–12^.

Nanoliposomal irinotecan and fluorouracil with folinic acid (NFF) is currently the standard regimen after GEM-based therapy for unresectable or recurrent PC (urPC) based on the results of the phase 3 (NAPOLI-1)^13^. In Japan, a randomized phase 2 trial (331,501 study)^14^ demonstrated significantly better progression-free survival (PFS) with NFF than with fluorouracil with folinic acid (FF) . However, only 40 patients were treated with NFF in the 331,501 study, and thus, we conducted this observational study to evaluate the efficacy and safety of NFF in a real-world setting (NAPOLEON-2 study). We report herein the results of the retrospective part of the NAPOLEON-2 study.

## Results

### Patient characteristics

**Figure 1:**
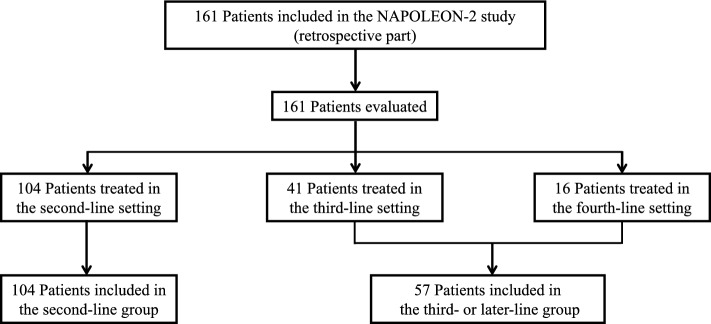
Flow diagram of the NAPOLEON-2 study (retrospective part).

A total of 161 patients with urPC who received NFF treatment were evaluated (Fig. 1). The median follow-up period was 7.3 months (95% confidence interval (CI), 5.6–8.9 months). The patient characteristics are shown in Table 1. The median patient age was 67 years (range, 38–85 years), and 88 patients (55%) were male. All patients had received GEM previously. Overall, 104 (65%) and 57 (35%) patients received NFF as second- or third- or later-line treatment, respectively. The median duration of prior chemotherapy was 9.8 months (range, 1.4–45.0 months). Three patients were homozygous for uridine diphosphate glucuronosyltransferase 1a1 (*UGT1A1*) *6, two were homozygous for UGT1A1*28, and three were heterozygous for UGT1A128* and *6.

**Table 1:** Patient characteristics (*n* = 161).

Characteristic		Value
Age, years	Median (range)	67 (38–85)
	≥ 75 years, *n* (%)	26 (16)
Sex, *n* (%)	Male	88 (55)
ECOG PS, *n* (%)	0	74 (46)
	1	76 (47)
	≥ 2	11 (7)
Stage, *n* (%)	Locally advanced	19 (12)
	Metastatic	142 (88)
History of pancreatomy, *n* (%)		40 (25)
History of radiation, *n* (%)		5 (3)
History of biliary drainage, *n* (%)		41 (26)
Duration time of previous chemotherapy, months	Median (range)	9.8 (1.4–45.0)
Treatment line of Nal-IRI, *n* (%)	2nd	104 (65)
	3rd or later	57 (35)
Previous drugs, *n* (%)	Gemcitabine	161 (100)
	Fluoropyrimidine	52 (32)
	Platinum	20 (12)
	Irinotecan	18 (11)
Histology, *n* (%)	Adenocarcinoma	148 (91)
	Others	7 (4)
	Uncertified	6 (4)
Tumor location, *n* (%)	Head	67 (42)
	Body/tail	94 (58)
Site of metastasis, *n* (%)	Liver	89 (55)
	Peritoneum	44 (27)
Ascites, *n* (%)	None	137 (85)
	Intrapelvic	17 (11)
	Extrapelvic	7 (4)
CA19-9, U/ml	Median (range)	1015 (1–543,522)
	≥ 37 U/ml, *n* (%)	130 (81)
NLR	Median (range)	3.16 (0.48–21.86)
	≥ 3.16, *n* (%)	81 (50)
CAR	Median (range)	0.079 (0.003–11.542)
	≥ 0.079, *n* (%)	79 (49)
UGT1A1, *n* (%)	Wild	80 (50)
	-/*6 or -/*28	64 (40)
	*6/*6, *6/*28 or *28/*28	8 (5)
	Unknown	9 (6)
Complication, *n* (%)	Any	94 (58)
	Diabetes	48 (30)
	Hypertension	37 (23)
	Cardiovascular disease	10 (6)
	Stroke	7 (4)

*Abbreviations: ECOG PS* Eastern Cooperative Oncology Group performance status, *Nal-IRI* nanoliposomal irinotecan, *CA19-9* carbohydrate antigen 19–9, *NLR* neutrophil-to-lymphocyte ratio, *CAR* C-reactive protein-albumin ratio, *UGT1A1* uridine diphosphate glucuronosyltransferase family 1 member A1.

### Treatment outcomes and safety

**Figure 2:**
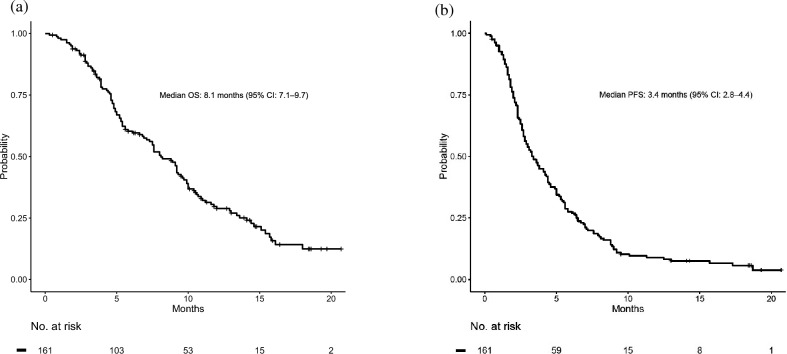
Kaplan–Meier survival curves for nanoliposomal irinotecan and fluorouracil with leucovorin. (**a**) Overall survival, (**b**) progression-free survival. *OS* overall survival*, CI* confidence interval*, PFS* progression-free survival.

**Figure 3:**
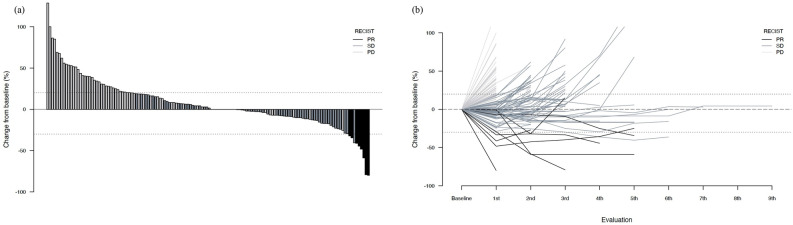
The response to treatment for NFF. (**a**) The response to treatment for NFF by Waterfall plot of maximum tumor shrinkage, (**b**) spider plot of treatment with NFF. *NFF* Nanoliposomal irinotecan and fluorouracil with folinic acid, *PR* partial response, *SD* stable disease, *PD* progressive disease.

The median OS (mOS) was 8.1 months (95% CI 7.1–9.7 months), and the median PFS (mPFS) was 3.4 months (95% CI 2.8–4.4 months) (Fig. 2). Figure 3a shows the response to treatment by waterfall plot. The objective response rate was 5%, and disease control rate was 52% (Table 2). The spider plot during the clinical course of NFF is shown in Fig. 3b. Table 3 shows the results with respect to treatment intensity. The median number of treatment cycles was 5 (range, 1–38). The initial doses and reasons for dose reduction during treatment are also summarized in Table 3. In total, 104 (65%) patients initially received a full dose of nanoliposomal irinotecan (nal-IRI). The median relative dose intensities (RDIs) for nal-IRI and 5-fluorouracil (5-FU) were 81.6% and 90.7%, respectively. The most common reason for dose-reduction during treatment was myelosuppression, followed by anorexia (Table 3).

**Table 2:** Response to NFF in all patients (*n* = 161).

Best overall response, *n* (%)	Value
CR	0
PR	8 (5)
SD	76 (47)
PD	64 (40)
NE	13 (8)
ORR (CR + PR), *n* (%)	8 (5)
DCR (CR + PR + SD), *n* (%)	84 (52)

*Abbreviations: NFF* Nanoliposomal irinotecan and fluorouracil with folinic acid, *CR* complete response, *PR* partial response, *SD* stable disease, *PD* progressive disease, *ORR* overall response rate, *DCR* disease control rate.

**Table 3:** Relative dose intensity and reasons for dose reduction of NFF.

Starting dose		Nal-IRI	Fluorouracil
Full dose	*n* (%)	104 (65)	123 (76)
Reduction level −1*	*n* (%)	51 (32)	30 (19)
Reduction level −2**	*n* (%)	6 (4)	8 (5)
Relative dose intensity	Median (range)	81.6 (53.0–105.7)	90.7 (40.8–108.3)
Treatment cycle	Median (range)	5 (1–38)	
Reasons for dose reduction during treatment, *n* (%)			
No reduction		94 (58)	99 (61)
Neutropenia		25 (16)	23 (14)
Anorexia		17 (11)	14 (9)
Nausea/vomiting		7 (4)	5 (3)
Diarrhea		3 (2)	5 (3)
Fatigue		2 (1)	2 (1)
Malaise		2 (1)	2 (1)
Others		10 (6)	10 (6)

*Reduction level −1: Nal-IRI 50 mg/m^2^, fluorouracil 1800 mg/m^2^.

**Reduction level −2: Nal-IRI 43 mg/m^2^, fluorouracil 1,350 mg/m^2^.

For patients homozygous of UGT1A1*6 or UGT1A1*28 or heterozygous of UGT1A1*6 and UGT1A1*28, the reduction level −1 for Nal-IRI is 43 mg/m^2^, and the reduction level −2 is 35 mg/m^2^.

*Abbreviations: NFF* Nanoliposomal irinotecan and fluorouracil with folinic acid, *Nal-IRI* nanoliposomal irinotecan, *UGT1A1* Uridine diphosphate glucuronosyltransferase Family 1 Member A1.

Treatment-related adverse events (AEs) affecting ≥ 10% of patients are summarized in Table 4. The most common AE was anorexia (73%), followed by general malaise (58%), and anemia (53%). Grade ≥ 3 neutropenia occurred in 38 patients (23%). Nonhematologic AEs of grade ≥ 3 included anorexia in 20 patients (12%); nausea in 9 (6%); general malaise, fatigue, and diarrhea in 5 (3%); vomiting and elevated liver enzymes in 2 (1%); and oral mucositis and peripheral neuropathy in 1 patient (1%). Febrile neutropenia was observed in one patient (1%).

**Table 4:** All-grade AEs occurring in ≥ 10% of all patients during NFF.

	All grades, *n* (%)	Grade ≥ 3, *n* (%)
Hematologic		
Neutropenia	68 (42)	38 (23)
Leukopenia	66 (41)	19 (12)
Anemia	86 (53)	15 (9)
Thrombocytopenia	20 (12)	2 (1)
Nonhematologic		
Anorexia	118 (73)	20 (12)
Malaise	94 (58)	5 (3)
Fatigue	79 (49)	5 (3)
Nausea	68 (42)	9 (6)
Vomiting	32 (20)	2 (1)
Diarrhea	51 (32)	5 (3)
Constipation	50 (31)	0 (0)
Liver enzyme elevation	41 (25)	2 (1)
Peripheral sensory neuropathy	63 (39)	1 (1)
Oral mucositis	20 (12)	1 (1)
Alopecia	36 (22)	0 (0)

*Abbreviations: AE* Adverse Event, *NFF* Nanoliposomal irinotecan and fluorouracil with folinic acid.

Multivariate analysis using the Cox regression analysis identified that shorter duration of previous chemotherapy, treatment line of NFF, ascites, higher neutrophil-to-lymphocyte ratio (NLR), and high C-reactive protein-to-albumin ratio were independent predictors of survival (Supplemental Table 1). At the final follow-up, 12 (7%) patients continued NFF treatment, and 1 patient (1%) withdrew due to transfer. NFF was discontinued in 148 patients (92%) due to worsening disease (*n* = 133), AEs (*n* = 13), and patient request (*n* = 2). Of them, 95 patients received best supportive care, and 53 patients did post-treatment.

### Additional analysis

**Figure 4:**
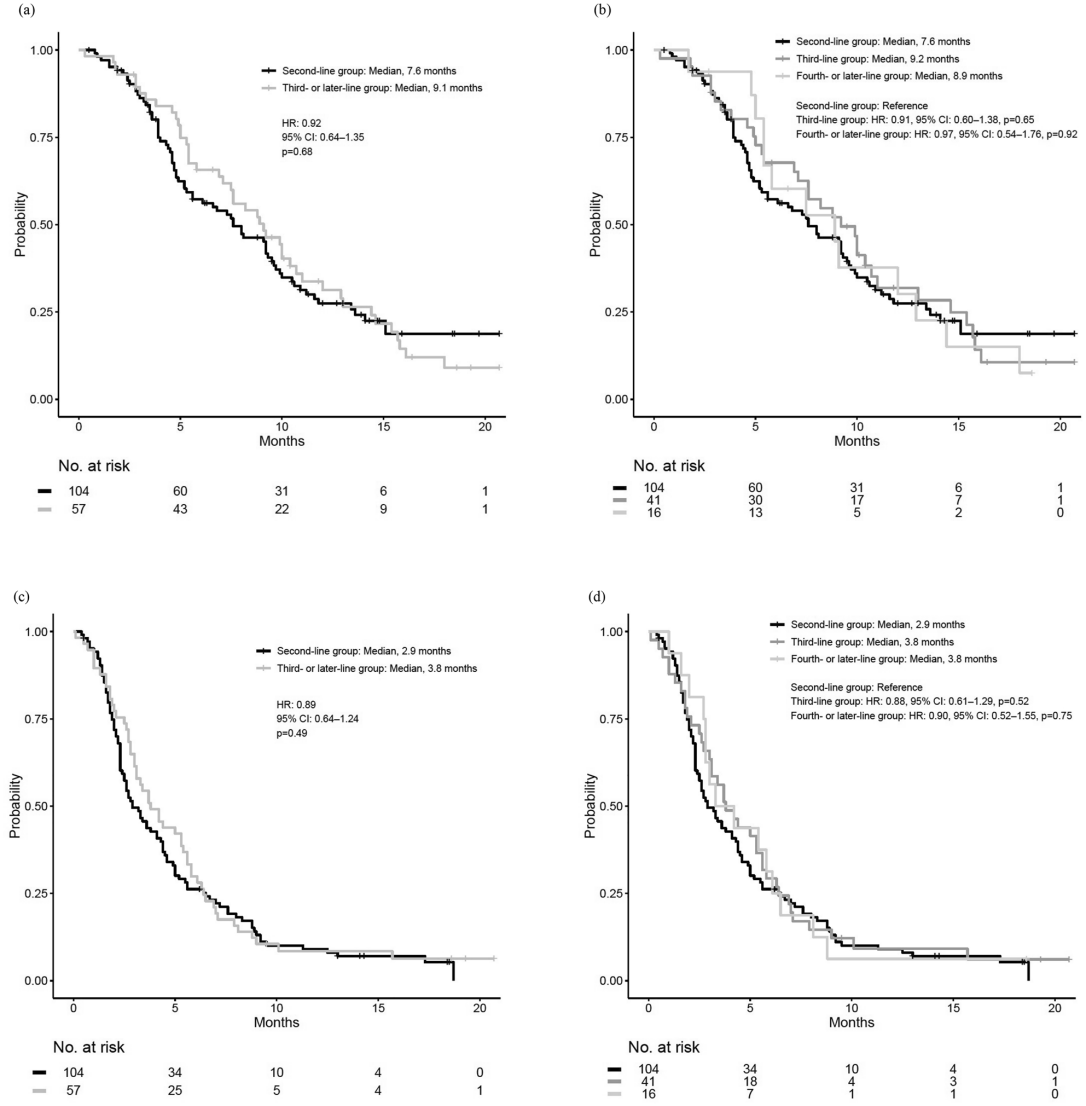
Kaplan–Meier survival curves of nanoliposomal irinotecan and fluorouracil with leucovorin in the second-line and third- or later-line groups. (**a**) Comparison of overall survival between the second-line and the third- or later-line groups, (**b**) Comparison of overall survival among the second-, third-, and fourth-or-later line groups, (**c**) Comparison of progression-free survival between the second-line and the third-or later-line groups, (**d**) Comparison of progression-free survival among the second-, third-, and fourth-or-later line groups. *HR* hazard ratio, *CI* confidence interval.

We categorized patients into two groups as the (a) second-line treatment group and (b) third-or-later-line treatment group (Fig. 1) and compared their clinical outcomes. The patient characteristics for this analysis are shown in Supplemental Table 2; there were significant differences in the groups. The median OS values of the second-line group and the third- or later-line group were 7.6 and 9.1 months, respectively (hazard ratio [HR]: 0.92; 95% CI 0.64–1.35; *p* = 0.68; Fig. 4a). For PFS, the median values of the second-line group and third- or later-line group were 2.9 and 3.8 months, respectively (HR: 0.89; 95% CI 0.64–1.24; *p* = 0.49; Fig. 4c). The objective response rates and disease control rates were not significantly different between the groups (Supplemental Table 3). The rate of treatment-related AEs affecting ≥ 5% of patients were also not significantly different between the two groups (Supplemental Table 4). Although the starting dose and minimum dose of fluorouracil tended to be lower in the third- or later-line group, those of nal-IRI were not significantly different (Supplemental Table 5).

In the third-or-later-line group, 41 and 16 patients were administered NFF as third- and fourth-or-later-line treatment, respectively (Supplemental Table 2). The median OS values in the second-line, third-line, and fourth-or-later-line treatment groups were 7.6, 9.2, and 8.9 months, respectively (Fig. 4b), and the median PFS values were 2.9, 3.8, and 3.8 months, respectively (Fig. 4d). Compared with the second-line group, the third-line and fourth-or-later-line groups did not show significant differences in both PFS (vs third-line group: HR: 0.88, 95% CI 0.61–1.29, *p* = 0.52; vs fourth- or later-line group: HR: 0.90, 95% CI 0.52–1.55, *p* = 0.75) and OS (vs third-line group: HR: 0.91, 95% CI 0.60–1.38, *p* = 0.65; vs fourth-or-later line group: HR: 0.97, 95% CI 0.54–1.76, *p* = 0.92). Additionally, in the third-or-later-line group, 18 patients received NFF after undergoing treatment with an irinotecan containing regimen, whereas 39 received NFF without prior exposure to an irinotecan containing regimen. The mOS values were 9.2 and 8.9 months, respectively (Supplementary Fig. 1a), and the median PFS values were 2.8 and 5.0 months, respectively (Supplementary Fig. 1b). No significant differences were observed in either OS and PFS. Patient characteristics and response to NFF are presented in Supplementary Table 6 and 7.

## Discussion

There have been limited clinical data on the usefulness and safety of NFF in the real-world setting. In the current study, the median OS and PFS of NFF were 8.1 and 3.4 months, respectively, which were more favorable than those reported in NAPOLI-1. The NAPOLI-1 study was the first prospective trial to demonstrate the efficacy of NFF for metastatic PC patients with refractory to GEM-based therapy^13^. The National Comprehensive Cancer Network guidelines also recommend NFF as a Category 1 regimen for second-or-later treatment and for recurrent cases^15^. In addition, NFF was generally well tolerated without any unexpected adverse events in the practical clinical setting. The median observation period was 7.3 months, which is shorter than the mOS in this study, but we consider this may have been influenced by the fact that the mOS for confirmed deaths was 5.6 months.

In the real-world application of regimens that have proven efficacy and safety in clinical trials, efficacy^16^ and treatment intensity are often reduced for safety reasons^17^. This is because clinical trials usually involve patients who are relatively young, in good general health, free from serious complications, and different from those in clinical practice^18^. The median age in the current study was higher than that in the NAPOLI-1 study (67 years vs. 63 years), and the proportion of patients with ECOG PS ≥ 1 was also higher (54% vs. 41%), However, we consider that these characteristics reflect the real-world. The proportions of patients with prior 5-FU and platinum were also lower, but there was no significant difference when compared with other clinical trials^13^. In addition, there was no significant difference in patient backgrounds compared to other real-world clinical observational studies^19–30^. Therefore, our study cohort is reasonable and similar with those in previous reports. The current sample size was 161 patients, and there have been a few reports presenting the efficacy of NFF with > 150 patients; thus, we consider that the study results are meaningful.

The mOS was 8.1 months, longer than that of the NAPOLI-1 study (median, 6.1 months) and of many retrospective and prospective studies^19–30^. This can be attributed to several reasons. First, the proportion of patients who had already received 5-FU in this study was slightly lower than that in others, and this might have contributed to a longer survival. Second, our study included some patients with locally advanced PC in the daily practice. Third, the proportion of patients receiving post-nal-IRI treatment was relatively higher (36%) than that in the NAPOLI-1 study (31%)^13^. Finally, the rate of AE-related treatment discontinuation was not high (8%) when compared with that in the NAPOLI-1 study (11%), and this might also have influenced the better OS. Given that AE is an important factor for continuing with subsequent chemotherapy, adequate management of AEs might help improve prognosis. In this aspect, approximately 40% of patients in the current study underwent dose reductions to reduce AEs. Dose reductions reportedly does not impact survival^22,25^, suggesting that appropriate dose modifications should be considered for longer-term continuation of treatment. We will also report the sub-analysis data on the RDI and dose modification in the future.

This study also compared the efficacy of NFF among the different treatment lines. There were no significant differences in PFS, OS, and safety between the second-line group and third-or-later-line groups, although some significant differences in patient backgrounds were observed. The third-or-later-line groups had significantly higher rates of prior treatments with FU, platinum, and irinotecan than had the second-line group; however, the third-or-later-line groups also included significantly younger patients and had lower rates of postoperative recurrence and liver metastases. Patients with postoperative recurrent PC have favorable prognosis, while liver metastases patients have poor prognosis^31–33^. Our study suggested that NFF might remain effective and safe even in the third-or-later-line setting if the general condition of the patients make them eligible for NFF^23,26^.

The safety profile of NFF in the current study appears acceptable in comparison with that in previous reports^19–30^. The incidence rates of grade 3 or 4 AEs were < 30%. Furthermore, grade 5 AEs were not observed. The most common grade 3 or 4 AEs was neutropenia (24%); however, febrile neutropenia occurred only in one patient (1%). Meanwhile, although grade 3 or 4 anorexia was only observed in 12% of the patients, this rate might still be slightly high. It is possible that the RDIs of nal-IRI and FU were higher than those in other reports^13,30^. Meanwhile, our study cohort included more patients who had undergone prior biliary drainage (26%) compared with the NAPOLI-1 trial cohort (13%)^13^. Our results suggest that NFF might be a safe treatment even in patients with prior biliary drainage, although further investigation is needed. In the subgroup analysis, the toxicity profile did not significantly differ between the second-line and third-or-later-line groups.

There were several limitations in this study. First, this is a retrospective observational study, which might have introduced selection bias and some difficulties in accurately assessing safety profiles. Therefore, we are now conducting a prospective study in another cohort, and the efficacy and safety will be validated. Second, this study included some patients who were clinically diagnosed with PC without histopathologic confirmation. Although histopathological diagnosis is recommended for patients undergoing chemotherapy^34^, there are some patients whose chemotherapy is initiated based on biochemical and/or radiological features only because of the difficult anatomical position of the pancreas or because of a clinical emergency. Third, NFF was administered for patients in both the second-line and third-or-later-line settings. Given that NFF is generally indicated for cases refractory to GEM, the treatment line of NFF in actual clinical practice is heterogeneous. However, we conducted a subgroup analysis and confirmed the safety and efficacy in the both the second-line and third-or-later-line settings.

In conclusion, NFF for PC is effective and safe in the second- and third-or later-line setting in the real-world. Our study is probably the most realistic observational study to date, as NFF was administered in patients with varying pretreatment histories, and efficacy and safety were retrospectively evaluated in a relatively large population.

## Methods

### Study design

The NAPOLEON-2 was a multicenter observational study comprised of a retrospective part and a prospective part to investigate the efficacy and safety of NFF as evaluated by specialists in gastroenterology and medical oncologists in Japan. The study population involved patients with urPC treated with NFF after previous GEM-based therapy. In the retrospective part, we reviewed the medical records of consecutive patients who received NFF for urPC at any of the 20 study hospitals between June 2020 and May 2021. Data on patient characteristics, treatment efficacy, and adverse events (AEs) were analyzed. Additionally, the survival efficacies of NFF were compared according to the treatment line, that is, between second-line and third- or later-line, and between after irinotecan containing regimen and not containing in third- or later-line.

This study was approved by each participating institution’s review board or ethics committee and was conducted according to the Declaration of Helsinki. Because this study was a retrospective observational study carried out in Japan, informed consent was obtained using the opt-in/opt-out approach according to each participating institution’s policy.

### NFF protocol

NFF was administered as follows: a 90-min intravenous (i.v.) infusion of nal-IRI (70 mg/m^2^), 46-h continuous i.v. infusion of 5-FU (2400 mg/m^2^), and 2-h i.v. infusion of levofolinate (200 mg/m^2^) every 2 weeks. At the discretion of the physician in charge, dose reduction at initiation and dose modification during treatment due to toxicities were allowed. Treatment was discontinued when disease progression or unacceptable AEs occurred even with dose adjustment, or at the patient’s request. Continuation of NFF treatment regimen after disease progression was permitted if deemed feasible by the physician.

### Assessments

The main endpoint of the retrospective part was OS. Other endpoints included the objective response rate, disease control rate, PFS, dose intensity, and AEs. Objective response and disease control were evaluated by computed tomography or magnetic resonance imaging according to the Response Evaluation Criteria in Solid Tumors (RECIST, version 1.1^35^). An objective response was defined as a complete or partial response, and disease control was defined as a complete or partial response with stable disease as the best response. OS was calculated from the date of administration of NFF to the date of death from any cause, or was censored at the final follow-up examination.

PFS was calculated from the date of administration of NFF to the date of progression or death from any cause, whichever was earlier, or was censored at the final follow-up examination. Treatment-related AEs were assessed according to the Common Terminology Criteria for Adverse Events, version 5.0^36^. Patient characteristics during NFF treatment were evaluated; these included age, sex, Eastern Cooperative Oncology Group performance status (ECOG PS), stage, medical history, previous chemotherapy information, histology, primary tumor site, metastatic site, ascites, carbohydrate antigen 19–9, NLR, C-reactive protein to albumin ratio, and *UGT1A1* status.

### Statistical analyses

The proportions and antitumor effects were compared using the Mann–Whitney test for continuous data and the χ2 test for categorical data. PFS and OS were estimated using the Kaplan–Meier method, and the probability of survival were compared using the log-rank test and the Cox proportional hazards model. Univariate and multivariate Cox proportional hazards models were used at the start of NFF treatment. The HR was expressed with 95% CI. Statistical significance was set at *p* < 0.05. All statistical analyses were performed using R version 4.2.2 (R Foundation for Statistical Computing, Vienna, Austria).

### Ethical approval and consent to participate

This study was conducted in accordance with the ethical guideline of the Declaration of Helsinki and was centrally approved by the Institutional review board of Sasebo Kyosai Hospital (study ID 2021–08), and also approved by the Institutional Review Boards or Ethics Committee of following institutions: Kagoshima City Hospital, National Cancer Center Hospital East, Kagoshima University Hospital, Kurume University Hospital, Saga Medical Center Koseikan, Japanese Red Cross Kumamoto Hospital, Karatsu Red Cross Hospital, Japan Community Healthcare Organization Kyushu Hospital, Japanese Red Cross Nagasaki Genbaku Hospital, Oita University Hospital, University of Miyazaki Hospital, National Hospital Organization Kumamoto Medical Center, Saiseikai Kumamoto Hospital, Kagoshima Kouseiren Hospital, Miyazaki Prefectural Miyazaki Hospital, Nagasaki University Hospital, Izumi General Medical Center, Asakura Medical Association Hospital, and Saiseikai Sendai Hospital prior to the study. Because this study was a retrospective observational study carried out in Japan, informed consent was obtained using the opt-in/opt-out approach according to each participating institution’s policy.

### Supplementary Information

Supplementary Information.

## Data Availability

All data generated or analyzed in this study are stored in a secured research database. Although they are not publicly available, they are available through the corresponding author upon reasonable request.

## Code availability

We used R ver. 4.2.2 (R Foundation for Statistical Computing, Vienna, Austria) for statistical analyses.
