# Supplementary material for: A multicenter retrospective observational NAPOLEON2 study of nanoliposomal irinotecan with fluorouracil and folinic acid in patients with unresectable pancreatic cancer
Source: Sci Rep. 2024 May 30;14:12422. doi: 10.1038/s41598-024-63172-y (PMC11139902; doi:10.1038/s41598-024-63172-y)
Supplement: Supplementary file 1 — Supplementary Information. [file 41598_2024_63172_MOESM1_ESM.pdf]

Supplement Figure 1

(a) (b)

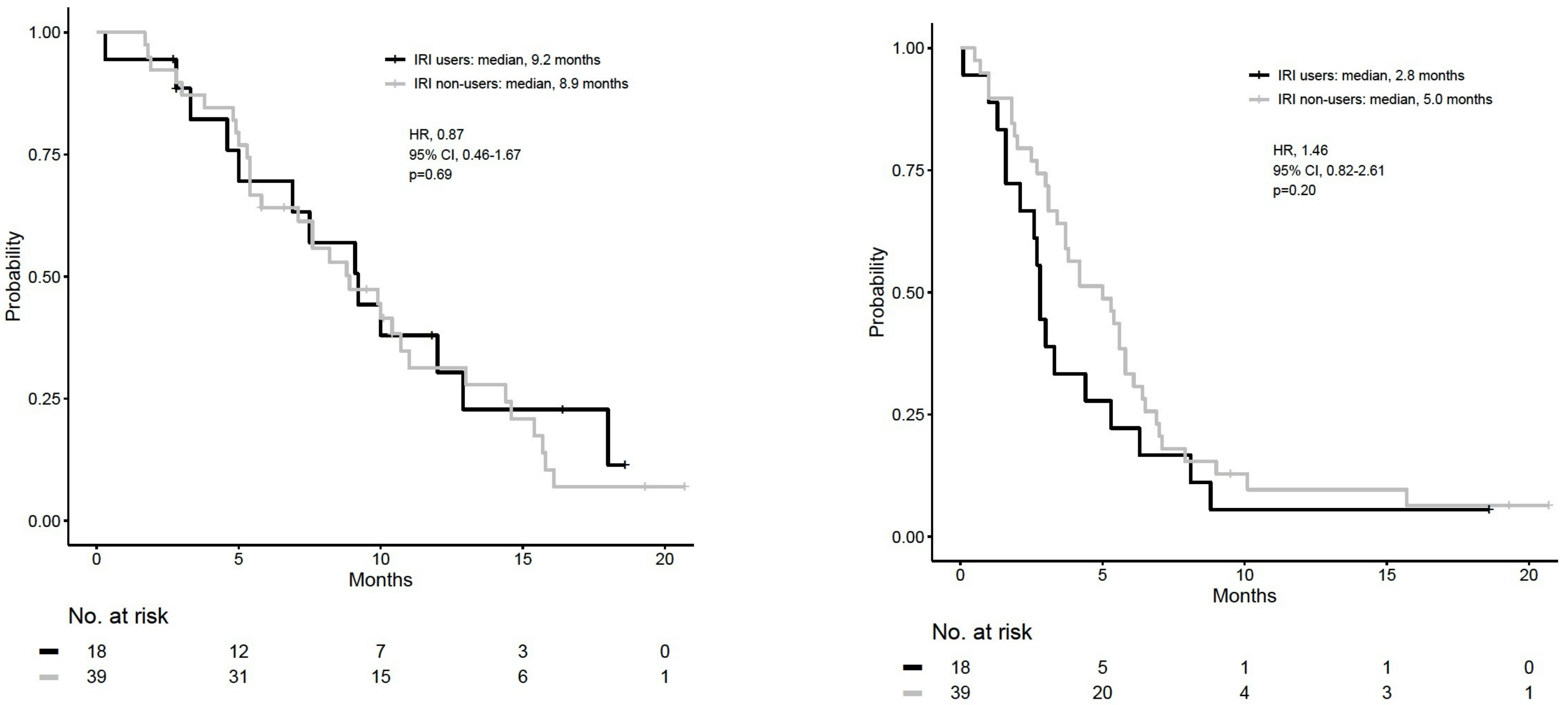

SF 1. Kaplan–Meier survival curves for nanoliposomal irinotecan and fluorouracil with leucovorin after irinotecan containing regimen and not containing regimen. (a) Overall survival, (b) progression-free survival.

HR hazard ratio, CI confidence interval, IRI users patients who had previously received irinotecan containing regimen, IRI non-users patients who had never received irinotecan containing regimen

Supplemental Figure legend

Kaplan–Meier survival curves for nanoliposomal irinotecan and fluorouracil with leucovorin after irinotecan containing regimen and not containing regimen. (a) Overall survival, (b) progression-free survival.

HR hazard ratio, *CI* confidence interval, *IRI users* patients who had previously received irinotecan containing regimen, *IRI non-users* patients who had never received irinotecan containing regimen

Supplemental Table 1. Univariate and multivariate Cox proportional hazards models to predict PFS and OS at the start of NFF

	PFS						OS					
	Univariate			Multivariate			Univariate			Multivariate		
	HR	95% CI	*p*-Value	HR	95% CI	*p*-Value	HR	95% CI	*p*-Value	HR	95% CI	*p*-Value
Age (years)												
≥75	0.79	0.51–1.22	0.29	0.99	0.61–1.61	0.96	0.68	0.41–1.14	0.15	0.78	0.45–1.36	0.38
ECOG PS												
≥1	1.13	0.82–1.56	0.45	0.98	0.69–1.41	0.92	1.38	0.95–1.99	0.09	1.12	0.74–1.68	0.59
History of pancreatomy												
yes	0.85	0.59–1.24	0.40	0.82	0.52–1.32	0.42	0.73	0.47–1.13	0.16	0.70	0.41–1.18	0.17
Duration of previous chemotherapy regimen (months)												
<9.8 (median)	1.48	1.06–2.07	0.02	1.62	1.08–2.43	0.02	1.64	1.13–2.39	0.01	2.38	1.48–3.83	<0.01
Treatment line of Nal-IRI												
3rd-or-later line	0.92	0.66–1.28	0.62	1.76	1.13–3.20	0.02	0.96	0.66–1.39	0.83	2.35	1.43–3.86	<0.01
Liver metastasis												
Yes	1.39	1.00–1.92	0.048	1.13	0.77–1.65	0.53	1.08	0.75–1.55	0.69	0.86	0.57–1.30	0.47

Ascites												
Yes	1.63	1.03–2.57	0.04	1.90	1.13–3.20	0.02	2.25	1.38–3.68	<0.01	2.52	1.45–4.40	<0.01
CA19-9 (U/mL)												
≥37.0 (upper limit of normal)	0.96	0.63–1.47	0.86	1.16	0.73–1.87	0.52	1.00	0.62–1.63	0.99	1.26	0.75–2.13	0.38
NLR												
≥3.16 (median)	1.36	0.98–1.88	0.06	1.14	0.79–1.63	0.49	1.84	1.27–2.67	<0.01	1.53	1.02–2.29	0.04
CAR												
≥0.079 (median)	2.21	1.58–3.10	<0.01	2.07	1.42–3.03	<0.01	3.03	2.06–4.46	<0.01	2.75	1.79–4.22	<0.01

Abbreviations: PFS progression-free survival, *OS* overall survival, *NFF* Nanoliposomal irinotecan and fluorouracil with folinic acid, *HR* hazard ratio, 95% CI; 95% confidence interval, *ECOG PS* Eastern Cooperative Oncology Group performance status, *Nal-IRI* nanoliposomal irinotecan, *CA19-9* carbohydrate antigen 19-9, *NLR* neutrophil-to-lymphocyte ratio, *CAR* C-reactive protein-albumin ratio

Supplemental Table 2. Patient characteristics by treatment line for NFF

Characteristic		Second-line group (n = 104)	Third- or later-line group (n = 57)	*p*-Value
Age, years	Median (range)	70 (40–82)	66 (38–85)	0.03
Sex, n (%)	Male	61 (59)	27 (47)	0.17
ECOG PS, n (%)	0	49 (47)	25 (44)	0.39
	1	50 (48)	26 (46)	
	≥2	5 (5)	6 (11)	
Stage, n (%)	Metastatic	92 (89)	50 (88)	0.89
History of pancreatomy, n (%)		14 (14)	26 (46)	<0.01
Duration time of previous chemotherapy, months	Median (range)	7.8 (1.4–33.0)	14.9 (4.9–45.0)	<0.01
Treatment line of Nal-IRI, n (%)	2nd	104 (100)	0	-
	3rd	0 (0)	41 (72)	
	4th or later	0 (0)	16 (28)	
Previous drugs, n (%)	Fluoropyrimidine	2 (2)	50 (88)	<0.01
	Platinum	0 (0)	20 (35)	<0.01
	Irinotecan	0 (0)	18 (32)	<0.01
Histology, n (%)	Adenocarcinoma	95 (91)	53 (93)	0.92
	Others	5 (5)	2 (4)	
	Uncertified	4 (4)	2 (4)	

Tumor location, n (%)	Head	37 (36)	30 (53)	0.04
Site of metastasis, n (%)	Liver	67 (64)	22 (39)	<0.01
	Peritoneum	29 (28)	15 (26)	0.83
Ascites, n (%)	+	18 (17)	6 (11)	0.25
CA19-9, U/ml	Median (range)	1252 (0.6–489500)	627 (0.6–543522)	0.33
UGT1A1, n (%)	*6/*6, *6/*28, *28/*28	5 (5)	3 (5)	0.9

Abbreviations: NFF Nanoliposomal irinotecan and fluorouracil with folinic acid, *ECOG PS* Eastern Cooperative Oncology Group performance status, *Nal-IRI* nanoliposomal irinotecan, *CA19-9* carbohydrate antigen 19-9, *UGT1A1* Uridine diphosphate glucuronosyltransferase family 1 member A1

Supplemental Table 3. Response to NFF by treatment line

Best overall response, n (%)	Second-line group (n = 104)	Third-or-later-line group (n = 57)	*p*-Value
CR	0	0	
PR	4 (4)	4 (7)	
SD	46 (44)	30 (52)	
PD	45 (43)	19 (33)	
NE	9 (9)	4 (7)	
ORR (CR+PR), n (%)	4 (4)	4 (7)	0.38
DCR (CR+PR+SD), n (%)	50 (48)	34 (60)	0.16

Abbreviations: NFF Nanoliposomal irinotecan and fluorouracil with folinic acid, *CR* complete response, *PR* partial response, *SD* stable disease, *PD* progressive disease, *NE* not evaluable, *ORR* overall response rate, *DCR*, disease control rate

Supplemental Table 4. AEs affecting ≥5% of patients during treatment with NFF in the second-line and third- or later-line groups

	All grades, n (%)		*p*-Value	Grade ≧3, n (%)		*p*-Value
	Second-line group (n = 104)	Third-or-later-line group (n = 57)		Second-line group (n = 104)	Third-or-later-line group (n = 57)	
Hematologic						
Neutropenia	40 (38)	28 (49)	0.19	22 (21)	16 (28)	0.32
Leukopenia	38 (37)	28 (49)	0.12	11 (11)	8 (14)	0.52
Anemia	60 (58)	26 (46)	0.14	10 (10)	5 (9)	0.86
Thrombocytopenia	11 (11)	9 (16)	0.96	1 (1)	1 (2)	0.66
Nonhematologic						
Anorexia	76 (73)	42 (74)	0.93	12 (12)	8 (14)	0.65
Malaise	60 (58)	34 (60)	0.81	3 (3)	2 (4)	0.83
Fatigue	48 (46)	31 (54)	0.32	2 (2)	3 (5)	0.24
Nausea	44 (42)	24 (42)	0.98	6 (6)	3 (5)	0.89
Diarrhea	29 (28)	22 (39)	0.16	4 (4)	1 (2)	0.46
Vomiting	19 (18)	13 (23)	0.49	1 (1)	1 (2)	0.66
Constipation	31 (30)	19 (33)	0.64	0 (0)	0 (0)	-
Liver enzyme elevation	24 (23)	17 (30)	0.35	2 (2)	0 (0)	0.29

Peripheral sensory neuropathy	41 (39)	22 (39)	0.92	1 (1)	0 (0)	0.46
Oral mucositis	11 (11)	9 (16)	0.34	0 (0)	1 (2)	0.18
Alopecia	23 (22)	13 (23)	0.92	-	-	-

Abbreviations: AE Adverse event, *NFF* Nanoliposomal irinotecan and fluorouracil with folinic acid

Supplemental Table 5. Administered dose and reasons for dose reduction or discontinuation of NFF in the second-line and third- or later-line groups

	Nal-IRI			Fluorouracil		
	Second-line group (n = 104)	Third-or-later-line group (n = 57)	*p*-Value	Second-line group (n = 104)	Third-or-later-line group (n = 57)	*p*-Value
Starting dose						
Full dose, n (%)	69 (66)	35 (61)	0.26	85 (82)	38 (67)	0.03
Reduction level 1, n (%)*	33 (32)	18(32)		17(16)	13(23)	
Reduction level 2, n (%)**	2 (2)	4 (7)		2 (2)	6 (11)	
Minimum dose during treatment						
Full dose, n (%)	33 (32)	16 (28)	0.31	51 (49)	17 (30)	<0.01
Reduction level 1, n (%) *	53 (51)	26 (46)		42 (40)	24 (42)	
Reduction level 2, n (%) **	17 (16)	12 (21)		11 (11)	14 (25)	
Reduction level 3, n (%) ***	1 (1)	3 (5)		0 (0)	2 (4)	
Reasons of reduction during treatment, n (%)						
No reduction	61 (59)	33 (58)		67 (64)	32 (56)	
Neutropenia	15 (14)	10 (18)		13 (13)	10 (18)	
Anorexia	15 (13)	2 (5)		9 (9)	5 (9)	
Nausea/Vomiting	12 (12)	5 (9)		4 (4)	1 (2)	

Diarrhea	1 (1)	1 (2)	2 (2)	3 (5)
Fatigue	1 (1)	1 (2)	1 (1)	1 (2)
Malaise	0 (0)	2 (4)	0 (0)	2 (4)
Anemia	1 (1)	0 (0)	1 (1)	0 (0)
Oral mucositis	0 (0)	1 (2)	0 (0)	1 (2)
Patient request	1 (1)	0 (0)	1 (1)	0 (0)
Unknown	0 (0)	1 (2)	0 (0)	1 (2)
Others	6 (6)	1 (2)	6 (6)	1 (2)

*Reduction level -1: Nal-IRI 50 mg/m^2, fluorouracil 1,800 mg/m^2, **reduction level -2: Nal-IRI 43 mg/m^2, fluorouracil 1,350 mg/m^2

***Reduction level -3: Nal-IRI 35 mg/m^2, fluorouracil 1,260mg/m^2

For patients homozygous for UGT1A1*6 or UGT1A1*28 or heterozygous for UGT1A1*6 and UGT1A1*28, the reduction level -1 for Nal-IRI is 43 mg/m^2, and the reduction level -2 is 35 mg/m^2.

Abbreviations: NFF Nanoliposomal irinotecan and fluorouracil with folinic acid, *Nal-IRI* nanoliposomal irinotecan, *UGT1A1* Uridine diphosphate glucuronosyltransferase family 1 member A1

Supplemental Table 6. Patient characteristics administered NFF after IRI use or non-use

Characteristic		IRI users (n = 18)	IRI non-users (n = 39)	*p*-Value
Age, years	Median (range)	64 (38–78)	66 (47–85)	0.08
Sex, n (%)	Male	11 (61)	16 (41)	0.16
ECOG PS, n (%)	0	8 (44)	17 (44)	0.19
	1	10 (56)	16 (41)	
	≥2	0 (0)	6 (15)	
Stage, n (%)	Metastatic	16 (89)	34 (87)	0.86
History of pancreatomy, n (%)		7 (39)	19 (49)	0.49
Duration time of previous chemotherapy, months	Median (range)	15.3 (6.8–45.0)	14.7 (4.9–36.2)	0.90
Treatment line of Nal-IRI, n (%)	2nd	0 (0)	0 (0)	-
	3rd	18 (100)	39 (100)	
Previous drugs, n (%)	Fluoropyrimidine	18 (100)	32 (82)	0.06
	Platinum	18 (100)	2 (5)	<0.01
	Irinotecan	18 (100)	0 (0)	<0.01
Histology, n (%)	Adenocarcinoma	15 (83)	38 (97)	0.09
	Others	2 (11)	0 (0)	
	Uncertified	1 (6)	1 (3)	
Tumor location, n (%)	Head	8 (44)	22 (56)	0.40
Site of metastasis, n (%)	Liver	8 (44)	14 (36)	0.54

	Peritoneum	4 (22)	11 (28)	0.63
Ascites, n (%)	+	2 (11)	4 (10)	0.92
CA19-9, U/ml	Median (range)	713 (2–543522)	561 (1–48499)	0.38
UGT1A1, n (%)	*6/*6, *6/*28, *28/*28	2 (11)	1 (3)	0.18

Abbreviations: NFF Nanoliposomal irinotecan and fluorouracil with folinic acid, *IRI* Irinotecan, *ECOG PS* Eastern Cooperative Oncology Group performance status, *Nal-IRI* nanoliposomal irinotecan, *CA19-9* carbohydrate antigen 19-9, *UGT1A1* Uridine diphosphate glucuronosyltransferase family 1 member A1

Supplemental Table 7. Response to NFF by IRI users or non-users

Best overall response, n (%)	IRI users, (n = 18)	IRI non-users, (n = 39)	*p*-Value
CR	0	0	
PR	1 (6)	3 (8)	
SD	7 (39)	23 (59)	
PD	9 (50)	10 (26)	
NE	1 (6)	3 (8)	
ORR (CR+PR), n (%)	1 (6)	3 (8)	0.77
DCR (CR+PR+SD), n (%)	8 (44)	26 (67)	0.11

Abbreviations: NFF Nanoliposomal irinotecan and fluorouracil with folinic acid, *IRI* Irinotecan, *CR* complete response, *PR* partial response, *SD* stable disease, *PD* progressive disease, *NE* not evaluable, *ORR* overall response rate, *DCR*, disease control rate
